# Supplementary material for: Sub-nanowatt microfluidic single-cell calorimetry
Source: Nat Commun. 2020 Jun 12;11:2982. doi: 10.1038/s41467-020-16697-5 (PMC7292832; doi:10.1038/s41467-020-16697-5)
Supplement: Supplementary file 2 — Supplementary Information [file 41467_2020_16697_MOESM2_ESM.pdf]

Supplementary Information

Sub-nanowatt Microfluidic Single-Cell Calorimetry

Hong et al.

Supplementary Note 1. Derivation of the fin equation to estimate thermal conductance of the calorimeter.

We consider half length of the suspend microfluidic tube (see Figure 1 in the main text), ranging from the base ($x = 0$) to the middle of the tube ($x = L$). The heat transfer equation within this domain can be written as:

$$\frac{d^2\theta(x)}{dx^2} - m^2\theta(x) = 0 \tag{1}$$

where $\theta(x) = T(x) - T_\infty$, $T(x)$ is the temperature along the tube, T_∞ is the ambient (or base) temperature, $m = \sqrt{\frac{hP}{kA_c}}$, h is the radiation heat transfer coefficient to the ambient, k is the effective thermal conductivity of the tube (including the water inside), A_c is the cross-sectional area of the tube, P is the outer perimeter of the tube.

The general solution for Equation (1) is:

$$\theta(x) = C_1 e^{mx} + C_2 e^{-mx} \tag{2}$$

The boundary conditions are:

a) at $x = 0$, $\theta(0) = 0$,

b) at $x = L$, $\frac{d\theta(x)}{dx} = \frac{q/2}{kA_c}$, where q is the total heat flux applied at $x = L$ (i.e.., middle of the tube).

Note that only half of this heat flux goes to either half of the tube.

With the boundary conditions, we can determine:

$$C_1 = \frac{q/2}{kA_c m(e^{mL} + e^{-mL})}, \tag{3}$$

$$C_2 = -\frac{q/2}{kA_c m(e^{mL} + e^{-mL})}. \tag{4}$$

Therefore, the analytical solution for the temperature profile of the suspended tube with a point heat source can be rearranged and expressed as:

$$\theta(x) = \left(\frac{q/2}{kA_c m}\right)\left(\frac{e^{mx} - e^{-mx}}{e^{mL} + e^{-mL}}\right). \tag{5}$$

Then, the temperature in the middle of the tube is

$$\theta_{\text{max}} = \theta(L) = \left(\frac{q/2}{kA_c m}\right)\left(\frac{e^{mL} - e^{-mL}}{e^{mL} + e^{-mL}}\right), \tag{6}$$

Finally, the thermal conductance of the entire tube (length = $2L$), defined as $G = q/\theta_{max}$, can be expressed as:

$$G = 2mkA_c\left(\frac{e^{mL} + e^{-mL}}{e^{mL} - e^{-mL}}\right). \tag{7}$$

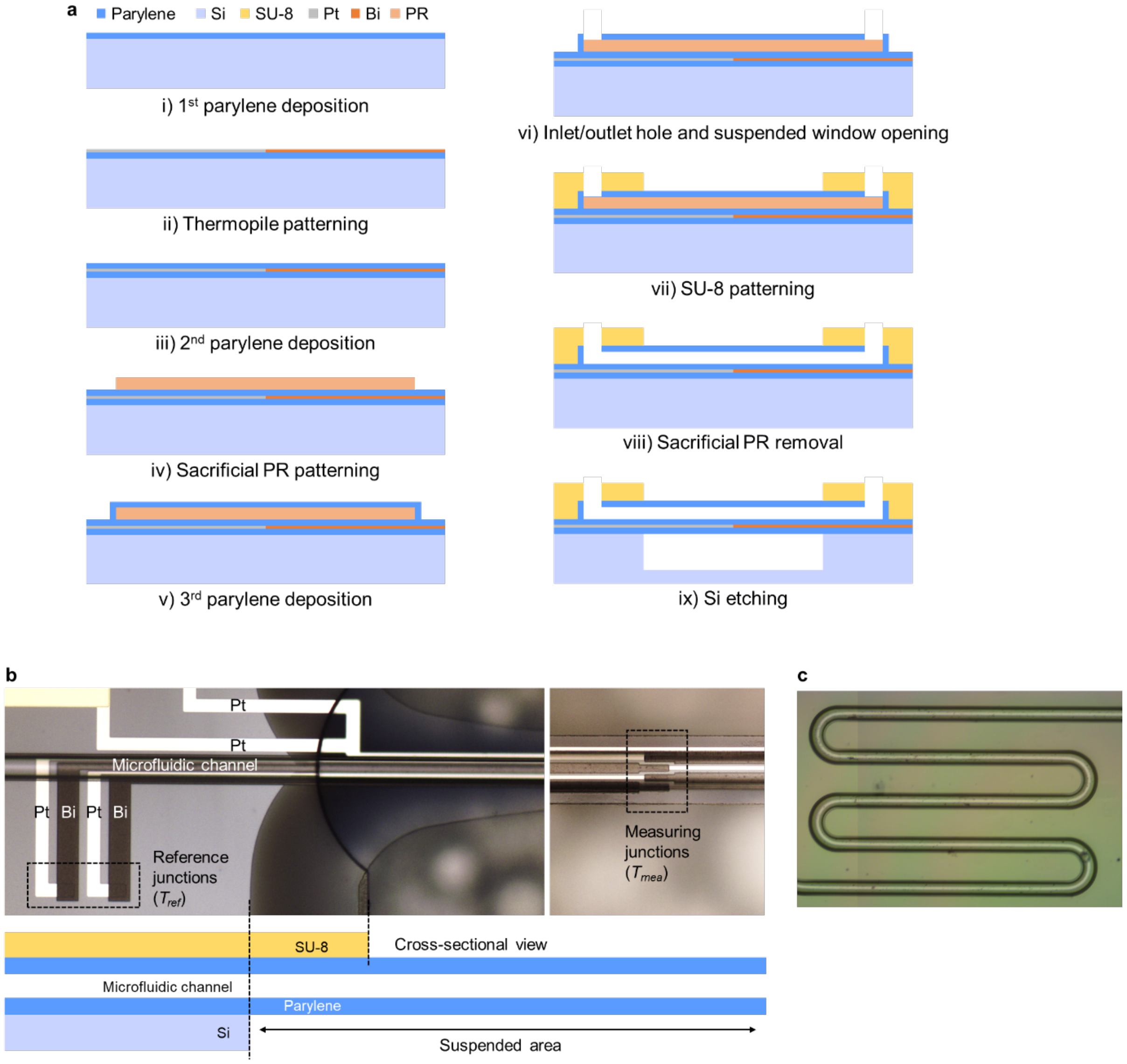

Supplementary Figure 1. Fabrication process and device design. a, Fabrication process of the calorimeter. **b,** Photographs of the calorimeter. **c,** Serpentine microfluidic channel on the temperature-controlled Si substrate to preheat the fluid and stabilize its temperature before it enters the suspended channel.

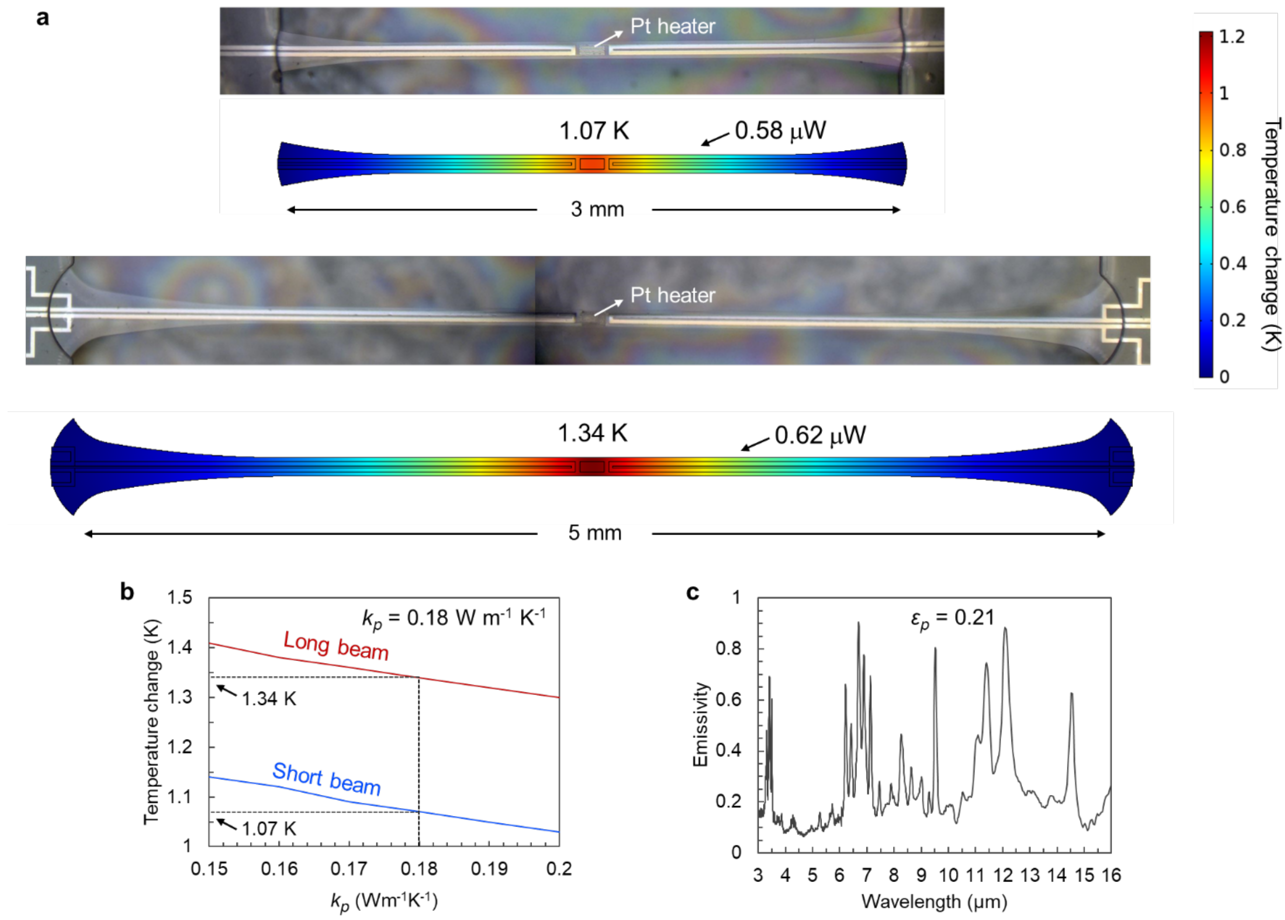

Supplementary Figure 2. Characterization of parylene properties. a, Photographs and COMSOL models to characterize the in-plane thermal conductivity of parylene. DC heating power was applied to the Pt heaters of both short and long parylene beams without microfluidic channel, and resultant temperature rise in the middle of the beams was measured by monitoring electrical resistance change of the Pt heater with AC current. **b,** COMSOL modeling result representing parylene thermal conductivity of 0.18 W m^{-1} K^{-1} matches well with the experimental results. **c,** Emissivity measurement of 12-μm thick parylene by FT-IR spectroscopy. The measured emissivity was 0.21 (weighted at 300 K).

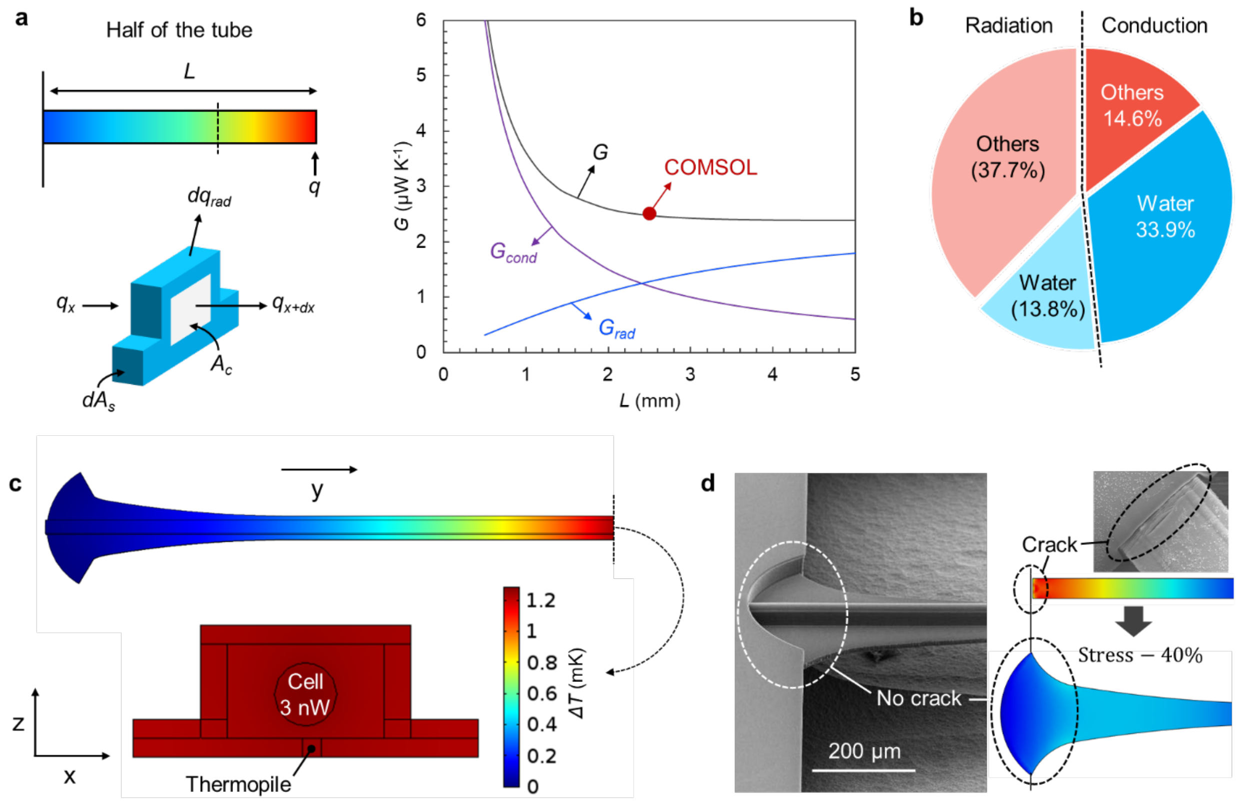

Supplementary Figure 3. Thermal and mechanical modeling of the calorimeter for design optimization. a, Estimation of overall thermal conductance (*G*) of the calorimeter. The fin equation (Equation (7)) was used for the design optimization. *G* saturates to 2.48 μW K^{-1} at 2.5 mm of the half tube length (*L*). COMSOL model reflecting the actual device geometry with a widened edge design also estimated similar overall thermal conductance (G = 2.51 μW K^{-1}) at *L* = 2.5 mm. **b,** Components that cause parasitic heat loss in the calorimeter. Radiation and conduction through water account for about a half of the total heat loss. **c,** The COMSOL model of the calorimeter. The simulation result shows that the temperature uniformity (the minimum temperature divided by the maximum temperature) within the cross-section of the suspended tube is more than 98%. This uniformity ensures that the temperature measured by the measuring junctions of the Bi-Pt thermopile represents the cell temperature regardless of the x- and z- position of the cell. **d,** Mechanical modeling to optimize interconnection design between the suspended and unsuspended parts. The widened edge design reduced the mechanical stress of the interconnection by 40%.

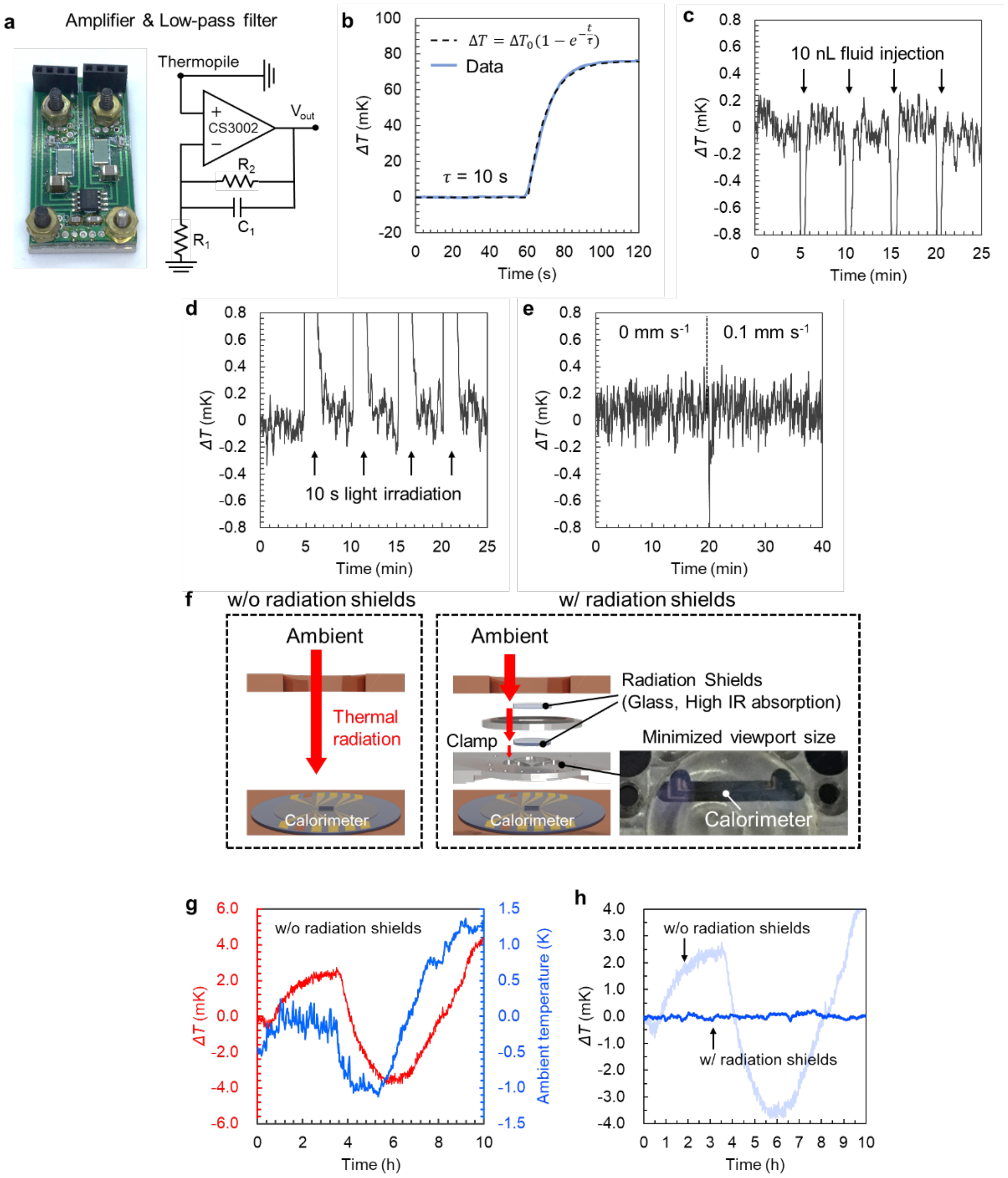

Supplementary Figure 4. Data acquisition and stability features of the calorimetry. a, Photograph and circuit diagram of operational amplifier and low-pass filter. **b,** Time constant of the calorimeter. **c,** Baseline stability with the series of fluid injections with high pulsed flow rate (~10 nL s^{-1}). **d,** Baseline stability with the series of light irradiation. **e,** Temperature stability with no flow and after applying continuous flow at 0.1 mm s^{-1} (corresponding to 0.175 nL s^{-1} flow rate from the syringe pump) **f,** Radiation shields to protect calorimeter from ambient thermal radiation. **g,** Temperature stability without thermal radiation shields. **h,** Temperature stability with thermal radiation shields. Source data for 4c, 4d, 4e, 4g, and 4h are provided as a Source Data file.

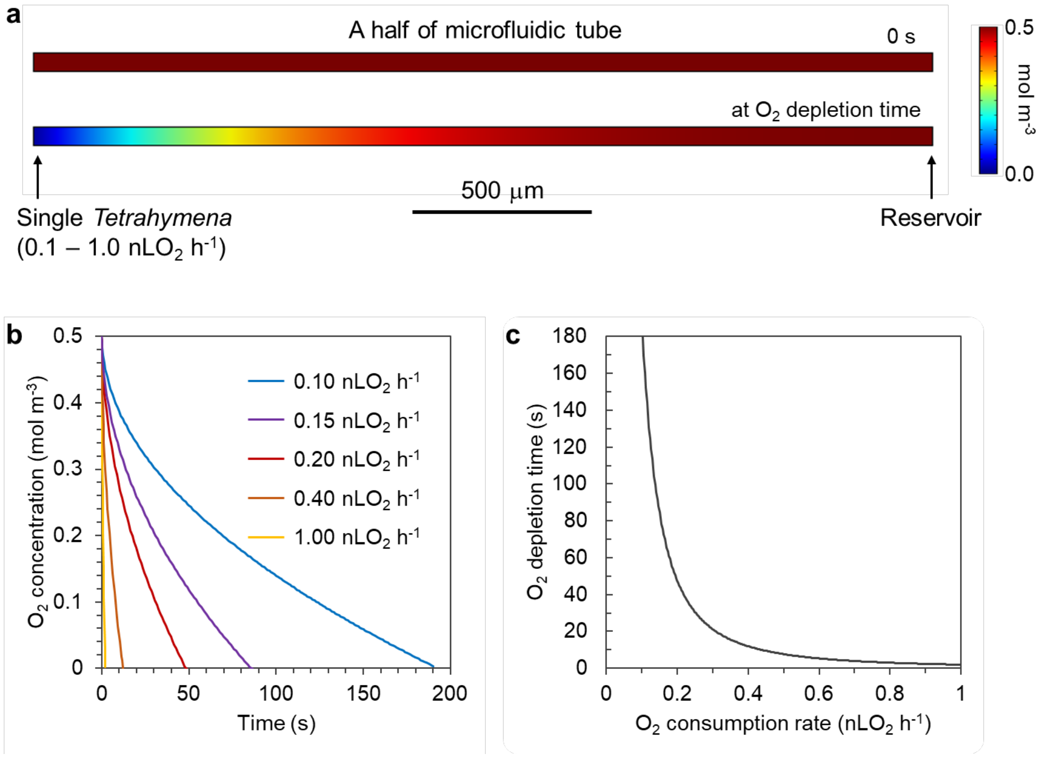

Supplementary Figure 5. Oxygen consumption of *Tetrahymena* in microfluidic channel without flow. a, COMSOL model of the oxygen consumption and diffusion, where oxygen solubility in water is 8 mg L^{-1}, oxygen consumption of *Tetrahymena* is 0.1–1.0 nLO_2 h^{-1} and oxygen diffusion rate in water is 2×10^{-5} cm^2 s^{-1}. **b,** Oxygen concentration near *Tetrahymena* as a function of time. **c,** Oxygen depletion time, when oxygen concentration reaches zero, as a function of oxygen consumption rate of *Tetrahymena*.

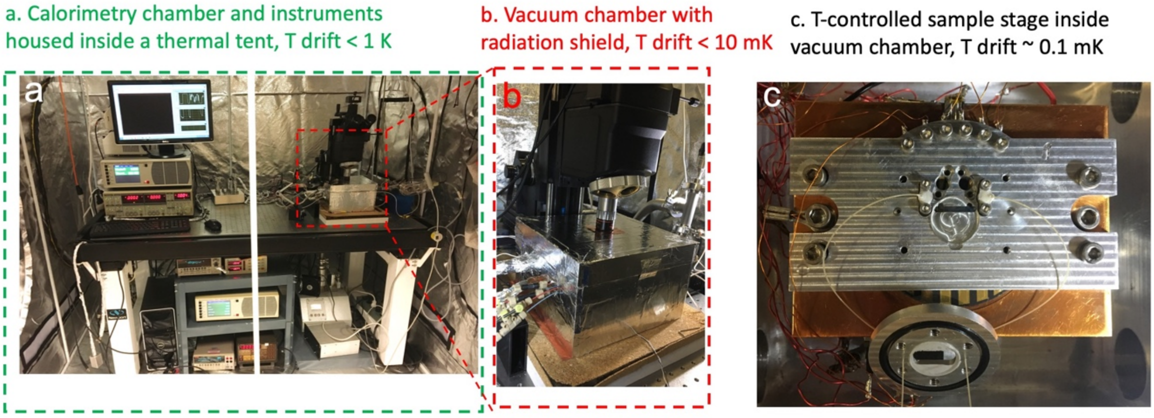

Supplementary Figure 6. Photographs of the experimental setup. (a) Calorimetry vacuum chamber (underneath the microscope, shown in the red box) and electronic instruments, all housed inside a temperature-controlled thermal tent with temperature drift less than 1 K. The instruments include two temperature controllers (Stanford Research Systems, PTC10), a lock-in amplifier (Stanford Research Systems, SR 830), a nanovoltmeter (Keithley 2182A), a turbo pump (Pfeiffer TMH 071P), A DC current source (Keithley 220). (b) Photography of the vacuum chamber with a radiation shield, inside which the microfluidic calorimeter is housed. The chamber is placed underneath a microscope for visualization. (c). Temperature-controlled Cu sample stage, on which a Si wafer with the suspended parylene microchannel is placed. An Al plated with fluidic in/out lets is used to clamp the Si wafer.

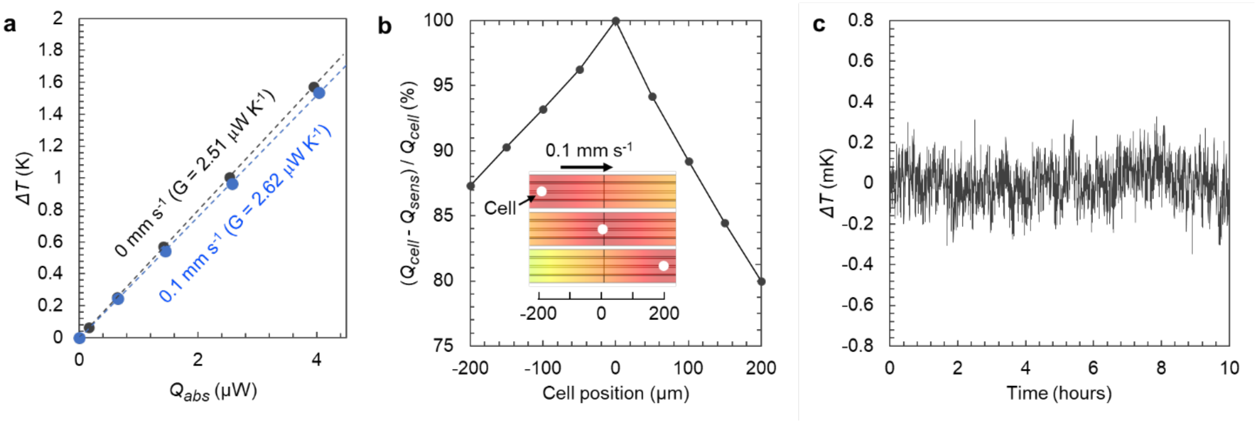

Supplementary Figure 7. Influence of fluid flow and cell position on the calorimeter sensitivity. **a,** Thermal conductance of the calorimeter with 0.1 mm s^{-1} of growth medium flow. **b,** Estimation of heat signal change depending on cell position. COMSOL model was used for the estimation. The result suggests that the measured heat signal by the calorimeter can be 20% lower than actual metabolic heat from a single cell when the cell is located 200 μm apart from the measuring junctions. Q_{cell} is the actual heat generation from the cell and Q_{sens} is the measured heat signal when the cell is located at different spots in the channel. "0" means the cells is located right on top of the junctions of the thermopile (or the middle of the microchannel). **c,** Temperature fluctuation of the microfluidic channel measured by the Pt/Bi thermopile for 10 h, under the influence of microscope illumination and 0.1 mm s^{-1} of growth medium flow. Source data for 7c and 7c are provided as a Source Data file.

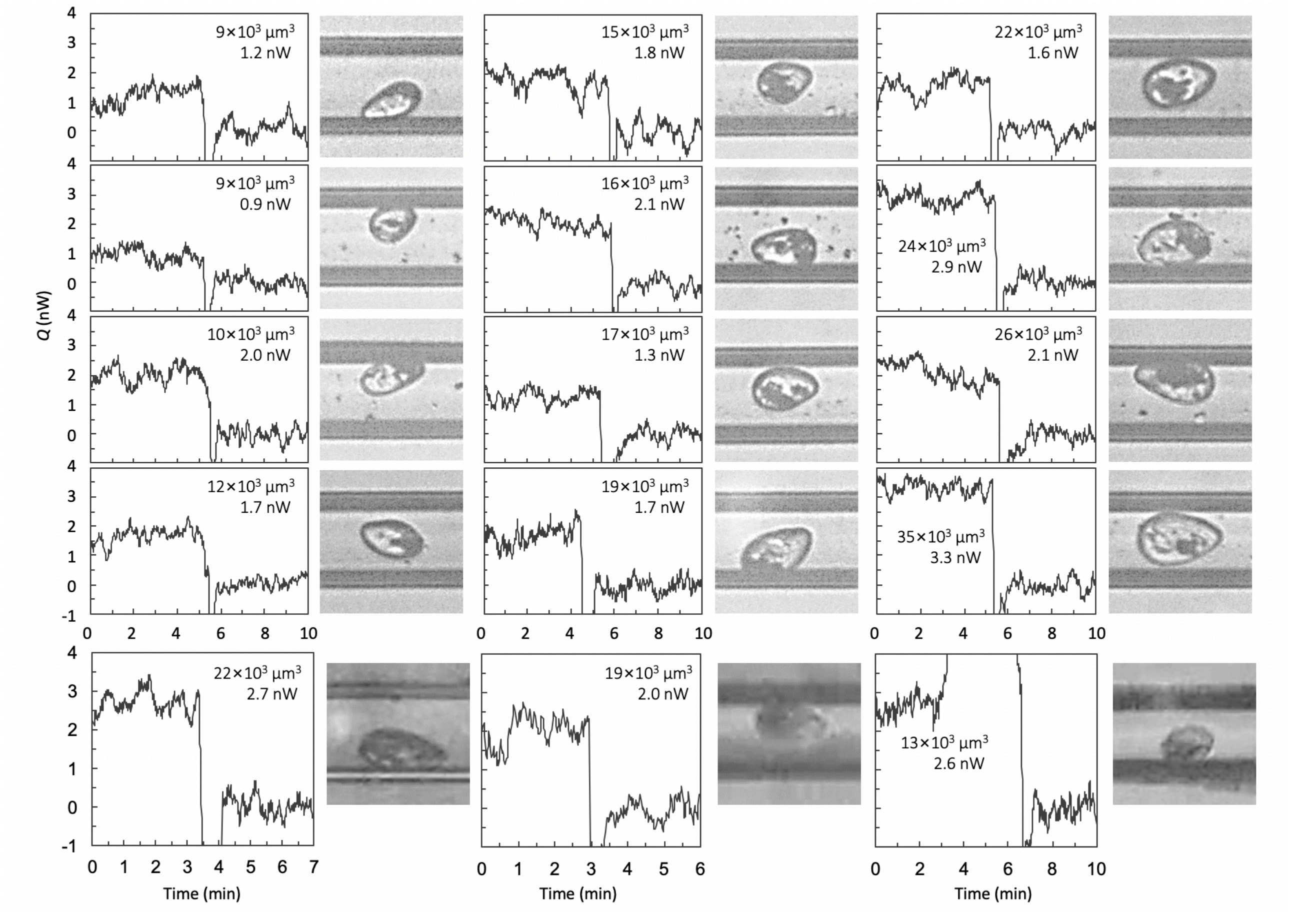

Supplementary Figure 8. Measured single-cell metabolic rates of *Tetrahymena* with various sizes. Source data for all the measurements shown in this figure are provided as a Source Data file.
