# Supplementary material for: Sub-nanowatt microfluidic single-cell calorimetry
Source: Nat Commun. 2020 Jun 12;11:2982. doi: 10.1038/s41467-020-16697-5 (PMC7292832; doi:10.1038/s41467-020-16697-5)
Supplement: Supplementary file 6 — Description of Additional Supplementary Files [file 41467_2020_16697_MOESM6_ESM.pdf]

Title: Supplementary Movie 1
Description: cell trapping

Title: Supplementary Movie 2
Description: cell injection

Title: Supplementary Movie 3
Description: cell removal
