# Supplementary material for: Sub-nanowatt microfluidic single-cell calorimetry
Source: Nat Commun. 2020 Jun 12;11:2982. doi: 10.1038/s41467-020-16697-5 (PMC7292832; doi:10.1038/s41467-020-16697-5)
Supplement: Supplementary file 7 — Reporting Summary [file 41467_2020_16697_MOESM7_ESM.pdf]

nature research

Corresponding author(s): Renkun Chen
Last updated by author(s): May 12, 2020

Reporting Summary

Nature Research wishes to improve the reproducibility of the work that we publish. This form provides structure for consistency and transparency in reporting. For further information on Nature Research policies, see Authors & Referees and the Editorial Policy Checklist .

Statistics

For all statistical analyses, confirm that the following items are present in the figure legend, table legend, main text, or Methods section.

n/a	Confirmed	
☐	☒	The exact sample size (*n*) for each experimental group/condition, given as a discrete number and unit of measurement
☐	☒	A statement on whether measurements were taken from distinct samples or whether the same sample was measured repeatedly
☒	☐	The statistical test(s) used AND whether they are one- or two-sided<br>*Only common tests should be described solely by name; describe more complex techniques in the Methods section.*
☒	☐	A description of all covariates tested
☒	☐	A description of any assumptions or corrections, such as tests of normality and adjustment for multiple comparisons
☐	☒	A full description of the statistical parameters including central tendency (e.g. means) or other basic estimates (e.g. regression coefficient) AND variation (e.g. standard deviation) or associated estimates of uncertainty (e.g. confidence intervals)
☒	☐	For null hypothesis testing, the test statistic (e.g. *F*, *t*, *r*) with confidence intervals, effect sizes, degrees of freedom and *P* value noted<br>*Give P values as exact values whenever suitable.*
☒	☐	For Bayesian analysis, information on the choice of priors and Markov chain Monte Carlo settings
☒	☐	For hierarchical and complex designs, identification of the appropriate level for tests and full reporting of outcomes
☒	☐	Estimates of effect sizes (e.g. Cohen's *d*, Pearson's *r*), indicating how they were calculated

Our web collection on statistics for biologists contains articles on many of the points above.

Software and code

Policy information about availability of computer code

Data collection	No software or code was used to collect the data
Data analysis	No special data analysis method was used in this study

For manuscripts utilizing custom algorithms or software that are central to the research but not yet described in published literature, software must be made available to editors/reviewers. We strongly encourage code deposition in a community repository (e.g. GitHub). See the Nature Research guidelines for submitting code & software for further information.

Data

Policy information about availability of data

All manuscripts must include a data availability statement. This statement should provide the following information, where applicable:

- Accession codes, unique identifiers, or web links for publicly available datasets
- A list of figures that have associated raw data
- A description of any restrictions on data availability

Source data for Figs 2a, 2b, 3f, 4(a-d) and Supplementary Figs. 4c, 4d, 4e, 4g, 4h, 7a, 7c, 8 have been provided as a Source Data file. All other data supporting the findings of this study are available from the corresponding author upon reasonable request.

Field-specific reporting

Please select the one below that is the best fit for your research. If you are not sure, read the appropriate sections before making your selection.

☒ Life sciences ☐ Behavioural & social sciences ☐ Ecological, evolutionary & environmental sciences

For a reference copy of the document with all sections, see nature.com/documents/nr-reporting-summary-flat.pdf

Life sciences study design

All studies must disclose on these points even when the disclosure is negative.

Sample size

We measured metabolic rates of individual tetrahymena cells. In total 15 individual cells were successfully measured (raw data in Supplementary Fig. 8). No sample size calculation was performed. The sample size was chosen so we had enough statistics to correlate the measured metabolic rate with the cells size (Fig. 4c).

Data exclusions

For single tetrahymena cell measurements, we included all the data that showed a positive heat generate rate (15 successful measurements, as shown in Supplementary Fig. 8 and Fig. 4), but we excluded data on cells that did not show any heat generation rate (as shown by no measured temperature change), which occur less than 20% of the time when a particular microfludic device was working properly. We believed these cells were dead and hence did not show any metabolic rate. This was further evidenced by our observation that these cells were not mobile in the fluidic channel.

Replication

We took a number of measures to confirm replication. First, the measured data were collected from multiple microfluidic devices (n>10, see Fig. 1) , over the course of this study. They all showed similar device characteristics. For measurements on the tetrahymena cells, we did 15 successful measurements on single cells as stated above. We did control experiments on dead cells (repeated n=4 times on separated experiments) and no cell (or just water, repeated n > 5 times on separated experiments), also shown in Fig. 4a. We also successfully measured multiple cells at a time (e.g., 2 and 3 cells, see Fig. 4a). The multiple-cell measurement was only done once, because it is difficult to manipulate the flow to have two similarly sized cells located at the center of the micro-channel.

Randomization

The individual tetrahymena cells were randomly chosen from each batch of the cultured cells.

Blinding

The investigators were not blinded to group allocation during data collection. The data (e.g., temperature change) were automatically collected with a computer upon the injection of the cells (and in some cases, injection of water only as a control). The data analysis was based on the calculation of the mean and the standard deviation of the collected temperature change data . Therefore, the data collection and the subsequent analysis are not sensitive to potential bias.

Reporting for specific materials, systems and methods

We require information from authors about some types of materials, experimental systems and methods used in many studies. Here, indicate whether each material, system or method listed is relevant to your study. If you are not sure if a list item applies to your research, read the appropriate section before selecting a response.

Materials & experimental systems

n/a	Involved in the study
☒	☐ Antibodies
☒	☐ Eukaryotic cell lines
☒	☐ Palaeontology
☒	☐ Animals and other organisms
☒	☐ Human research participants
☒	☐ Clinical data

Methods

n/a	Involved in the study
☒	☐ ChIP-seq
☒	☐ Flow cytometry
☒	☐ MRI-based neuroimaging
